# Mapping Fishing Effort through AIS Data

**DOI:** 10.1371/journal.pone.0130746

**Published:** 2015-06-22

**Authors:** Fabrizio Natale, Maurizio Gibin, Alfredo Alessandrini, Michele Vespe, Anton Paulrud

**Affiliations:** 1 European Commission Joint Research Centre Institute for the Protection and Security of the Citizen, Ispra, Italy; 2 Swedish Agency for Marine and Water management, Göteborg, Sweden; Hellenic Centre for Marine Research, GREECE

## Abstract

Several research initiatives have been undertaken to map fishing effort at high spatial resolution using the Vessel Monitoring System (VMS). An alternative to the VMS is represented by the Automatic Identification System (AIS), which in the EU became compulsory in May 2014 for all fishing vessels of length above 15 meters. The aim of this paper is to assess the uptake of the AIS in the EU fishing fleet and the feasibility of producing a map of fishing effort with high spatial and temporal resolution at European scale. After analysing a large AIS dataset for the period January-August 2014 and covering most of the EU waters, we show that AIS was adopted by around 75% of EU fishing vessels above 15 meters of length. Using the Swedish fleet as a case study, we developed a method to identify fishing activity based on the analysis of individual vessels’ speed profiles and produce a high resolution map of fishing effort based on AIS data. The method was validated using detailed logbook data and proved to be sufficiently accurate and computationally efficient to identify fishing grounds and effort in the case of trawlers, which represent the largest portion of the EU fishing fleet above 15 meters of length. Issues still to be addressed before extending the exercise to the entire EU fleet are the assessment of coverage levels of the AIS data for all EU waters and the identification of fishing activity in the case of vessels other than trawlers.

## Introduction

Fisheries research in the EU is heavily relying on effort, catch and fleet capacity data from the fleet register, the logbooks, the sales notes and the Vessel Monitoring System (VMS) established by the control regulation (Council Regulation (EC) No 1224/2009).

While VMS data provides detailed information on the vessel tracks at high spatial temporal resolution, the logbooks include essential information on the gear used, species and volume of the catches.

The availability of VMS data has been indicated as a “revolution” for fisheries research [1]. VMS information gives the opportunity to assess more precisely the impacts of fishing activity in space. In addition, VMS allows improving time precision of effort estimates moving from a resolution of 24 hours or calendar day (normally adopted when processing logbook data) to the 2 hours intervals of VMS messages which represents the typical transmission frequency for VMS.

Several studies have proved the value of using effort data at these finer time and space resolutions when evaluating environmental impacts of fishing activity [2, 3, 4]. A not yet fully developed area of application is in fisheries economic studies where only few papers have been published until now about spatially explicit bio-economic models [5, 6]. With precise spatial information on fishing behaviour there are new possibilities to disaggregate economics at the level of coastal communities [7], exploring dependencies between coastal communities and fishing grounds and derive quantitative analyses and agent based models of fishing behaviour.

The use of VMS data poses a series of data management and methodological challenges linked to the treatment of large volumes of data and the need to relate tracking data to fishing activity. Two main software libraries, VMStools [8] and VMSbase [1], have been developed in the R statistical language to process and analyse VMS and logbook data. Both libraries provide functionalities for cleaning the data, interpolating between consecutive VMS messages, merging VMS and logbook data, clustering the fleet into métier, discriminating between fishing and not fishing activity and producing high resolution maps of fishing effort.

Methods for the interpolation of consecutive VMS messages range from a simple straight line interpolation to more sophisticated approaches like a cubic Hermite spline interpolation [9] or incorporating specific variables which have an influence on vessel navigation such as human control and drift [10].

Most of the methods developed for the discrimination between fishing and non-fishing activity rely on the analysis of speed profiles [11], either through statistical and data mining approaches or through the reference to thresholds based on expert knowledge. The frequency distributions of the speed profiles of fishing vessels with towed gear typically show a bimodal shape with the first mode, at lower speed, corresponding to the fishing activity and the second mode, at higher speed, corresponding to steaming. The statistical methods for the analysis of speed profiles available in the package VMStools are based on a segmented regression of the cumulative frequencies of speed data of individual vessels or of groups of vessels [8]. More complex classification methods use also information on the bearing in combination with Bayesian approaches [12, 13, 14].

Despite the promising methodological developments and increasing number of applications and tools, the extensive use of VMS data for scientific purposes is hindered by the difficulty of accessing control data for scientific purposes [15], often due to confidentiality and personal data protection concerns. The need to negotiate the access to VMS data with national control authorities has confined so far the research applications to specific fisheries and local areas.

In addition to the VMS, another system providing detailed vessel positioning data is the Automatic Identification System (AIS). AIS was introduced by the International Maritime Organisation (IMO) to improve maritime safety and avoid ship collisions [16]. Differently from VMS, which is usually based on point-to-point satellite communications between the ship and the ground centres, AIS messages are broadcasted by the vessels omni-directionally and can be received by other ships in the neighbourhood, by ground based receivers and by satellites. The AIS system provides the possibility for ships to exchange in near real time state vector (position, speed, course, rate of turn etc.), static (vessel identifiers, dimensions, ship-type etc.) and voyage related information (destination, ETA, draught etc.) [17]. The wealth of information that can be collected from AIS is progressively making such system a cornerstone of maritime surveillance not only for safety but also for security and situational awareness applications [18].

The AIS has been progressively extended in the EU to medium-large size fishing vessels and has become compulsory since May 2014 for all fishing vessels of more than 15 meters of length (EU Dir 2011/15/EU). To our knowledge there are yearly controls in some EU Member States that AIS is installed and functioning but there are no systematic controls if the system is used. Regardless of the level of compliance the use of AIS in the fishing sector has recently generated interest of researchers in the fisheries domain for its potential to map fishing activities [19].

A not trivial advantage offered by the AIS in respect of the VMS lies in the time resolution of the AIS messages with respect to the 2 hours of VMS. Depending on the specific manoeuvre of the vessel, AIS messages transmission rates range from 2 seconds (high speed or rate of turn) to 3 minutes (vessel at anchor). On the other hand, being AIS originally conceived for collision avoidance, the spatial coverage is limited to line-of-sight or, in specific cases, to VHF ducting propagation. This means that AIS messages are nominally received at distances in the order of a few to 100 natutical miles depending on the transmitter and receiver heights. AIS messages can be received also from Low Earth Orbit (LEO) satellites; however the refresh rate in this case is lowered to the satellite revisit time. Moreover, in high traffic density areas there is the problem of message collisions with the result of making the satellite receiver almost “blind” in such areas. By all means, spatial coverage maps have to be derived and associated to surveillance means in order to mitigate the ambiguities related to the actual absence of ships (true negatives) or lack of receiver coverage (false negatives).

The availability of AIS data through public web platforms or commercial services has raised several confidentiality and personal data protection concerns. The IMO has deprecated the publication for commercial purposes on the internet or elsewhere of AIS data transmitted by ships and indicated that the publication of such data could be detrimental to the safety and security of ships and port facilities [20]. In 2012 the European Data Protection Supervisor issued an opinion on the use of AIS and VMS data [21]. The opinion states that as long as the data can be linked to identified or identifiable individuals it entails the treatment of personal information. Under such circumstances the treatment of the data should follow a general principle of “limitation of purpose” and be confined to general law enforcement and objectives connected with the Common Fishery Policy.

The concerns on the confidentiality of VMS and AIS underlie the need to treat the data preserving anonymity and to present the research results at a level of aggregation which does not allow the linking to individual vessels, enterprises or individuals.

While respecting these requirements, the availability of public AIS data may be seen as an alternative to VMS data, for scientific analyses. The fact that AIS data is not related to control purposes and exchanged also in public domains expands its availability in respect of the VMS and offers a unique opportunity not only to analyse fishing behaviour at very detailed level but also to extend the analysis at supra-national level and outside the EU. At the more technical level the higher refresh rate of AIS messages solves the need of interpolating positions between consecutive messages, while posing higher computational demands given the higher volume of data.

Since AIS is designed with safety rather than control in mind it does not offer necessarily a best alternative in respect of VMS for control of illegal fishing and enforcement. In particular the fact that the system does not entail point to point transmission to control centres implies that the level of coverage may be discontinuous and not robust enough for a systematic control of individual vessels behaviour.

In this paper we explore the possibility of using AIS data for fisheries research and provide an analysis about the level of uptake of the AIS by the EU fishing fleet. This is part of a more long term objective of producing a high resolution map of fishing effort for Europe using AIS.

Issues to be addressed before extending the exercise to the entire EU fleet are the assessment of the coverage level of AIS signals for all EU waters and the refinement of the methodology for the identification of fishing activity also for fisheries and individual vessels where speed profiles do not exhibit the typical bimodal distributions of trawlers.

## Data and Methods

Our analysis is structured in the following 5 main steps: (1) we match the AIS global dataset with the EU fleet register to evaluate the uptake of the AIS system for the entire EU fleet and at port level; (2) we assess the level of coverage of AIS signals; (3) we develop a method for the identification of fishing activity which can be adapted to the higher computational demands posed by the larger AIS datasets; (4) we validate this method using logbook data; (5) we provide a first example of high resolution map of fishing effort and compare it with a similar map derived from logbook data.

The level of uptake of AIS in the EU fleet was estimated combining the terrestrial AIS dataset from the US Maritime Safety and Security Information System network with other data sources in the Blue Hub research platform maintained at the Joint Research Centre [22]. The vessel position data covered a time period from January 2014 to August 2014. The amount of data processed and analysed was of about 200 million unique AIS messages per month collected at global scale from 130,000 different vessels of all categories. The steps 2 to 5 were performed using the Swedish fishing fleet as cases study. The dataset included in this case around three million records from 177 fishing vessels.

### Linking between the AIS dataset and the fleet register

A join algorithm was developed to link the fishing vessels in the EU fishing fleet register to the global AIS dataset. The matching was performed using the call sign identifier, which is assigned to any radio transmission station. The joining using the call sign identifier as key produced a rate of matching of 70%. The mismatch could be attributed to absence of the call sign identifier in the EU fleet register (about 3.8% of the fishing vessels), misspelling of the identifier, or vessels not present in the AIS dataset (lack of AIS coverage, not operative vessels, AIS not used). In order to solve problems of misspelling a matching algorithm was implemented using the Levenshtein and Jaro strings matching distances functions [23]. The two functions were applied in two steps to find matches in vessel names and callsign identifiers. The match was considered successful if the distance was above a specified threshold and a one to one match was achieved. After the processing with the matching algorithms, the rate of matching between the EU fishing fleet register and the AIS dataset was improved to 80% of all the registered vessels of more than 15 m in length.

### Assessment of coverage

AIS signals analysed in this study are collected by a network of ground based receivers. As a consequence, the reception capabilities are subject to VHF propagation conditions, often related to line-of-sight between the transmitter (AIS on board the vessel) and the receiving station (ground based receiver). A coverage analysis of the AIS signal is thus necessary to understand the extent to which historical data can be used for statistical analysis, ultimately leading to fishing effort maps. In other words, it is necessary to estimate areas where we expect good spatial reception and identify areas where the network has insufficient or no coverage capabilities. In the latter case, any statistical analysis would result in underestimated figures.

The AIS signal coverage map was produced for the fishing area of the Swedish fleet. An algorithm was implemented to compute the trajectories from the AIS observations related to the same vessels. For each vessel identifier, the corresponding set of AIS positions were isolated and were separated in several subsets based on a time and geographical distance threshold. After this first step for each subset, the AIS positions were interpolated into unique trajectories. The density of trajectories was then computed based on a geographical grid at a spatial resolution of 0.1 degrees latitude by 0.1 degrees longitude. For each cell, the AIS position and the interpolated trajectory densities were computed and compared. The ratio between the AIS position and the trajectory interpolated points was used to estimate the AIS spatial coverage capabilities. For instance, areas showing a high trajectory density and therefore traffic and a number of AIS messages lower than expected were considered with poor AIS spatial coverage.

### Identification of fishing activity

The methodology used for the identification of fishing activity is based on assuming a fishing behaviour highly dependent and characterised by speed. Detecting changes and frequency of speed will help identifying which part of the vessel track can be considered as fishing and which not. Among the different fishing behaviours, those vessels employing a mobile gear are characterized by a clear speed fingerprint. Hence we will expect better parameters estimates for trawlers.

GIS techniques were abandoned since they are computational intensive [24] and not very efficient (*O*(*n* ^2^) or more). The size of the dataset used shifted our approach from a model based one to a data driven one. After performing the initial routine conversion from data types we applied some cleaning filters on the data. First of all we considered only those messages where the speed was greater than 0.5 knots. This filter resulted in a significantly smaller dataset, from 3 million to 1 million records. This choice was justified by an exploratory data analysis of the AIS messages showing a concentration of low speed messages in the proximity of the ports.

Some of the vessels had very few messages indicating that the system was present but not systematically used. The vessels considered were finally reduced to 156 after selecting those with more than 300 received messages. This threshold is suggested by Wengrzik as a robust number of observations providing robust Gaussian distributions parameters estimations through the EM algorithm [25]. A final cleaning filter consisted in omitting outliers and probable errors represented by messages whose speed was higher than the upper quartile plus 1.5 times the interquartile range.

After the data preparation process we investigated the distribution of every vessel speed profile by creating histograms of the speed. The speed histograms of most vessels showed a bimodal distribution, with the two modes corresponding to steaming (high speed) and fishing (low speed) behaviours. The bimodal distribution of speeds can be interpreted as the mixture of two Gaussian curves. The core of our methodology is based on the detection of the parameters of the Gaussian distributions. This represents an improvement over the preliminary work presented in [19], where the fishing behaviour was identified on the basis of domain expert knowledge.

**Fig 1:**
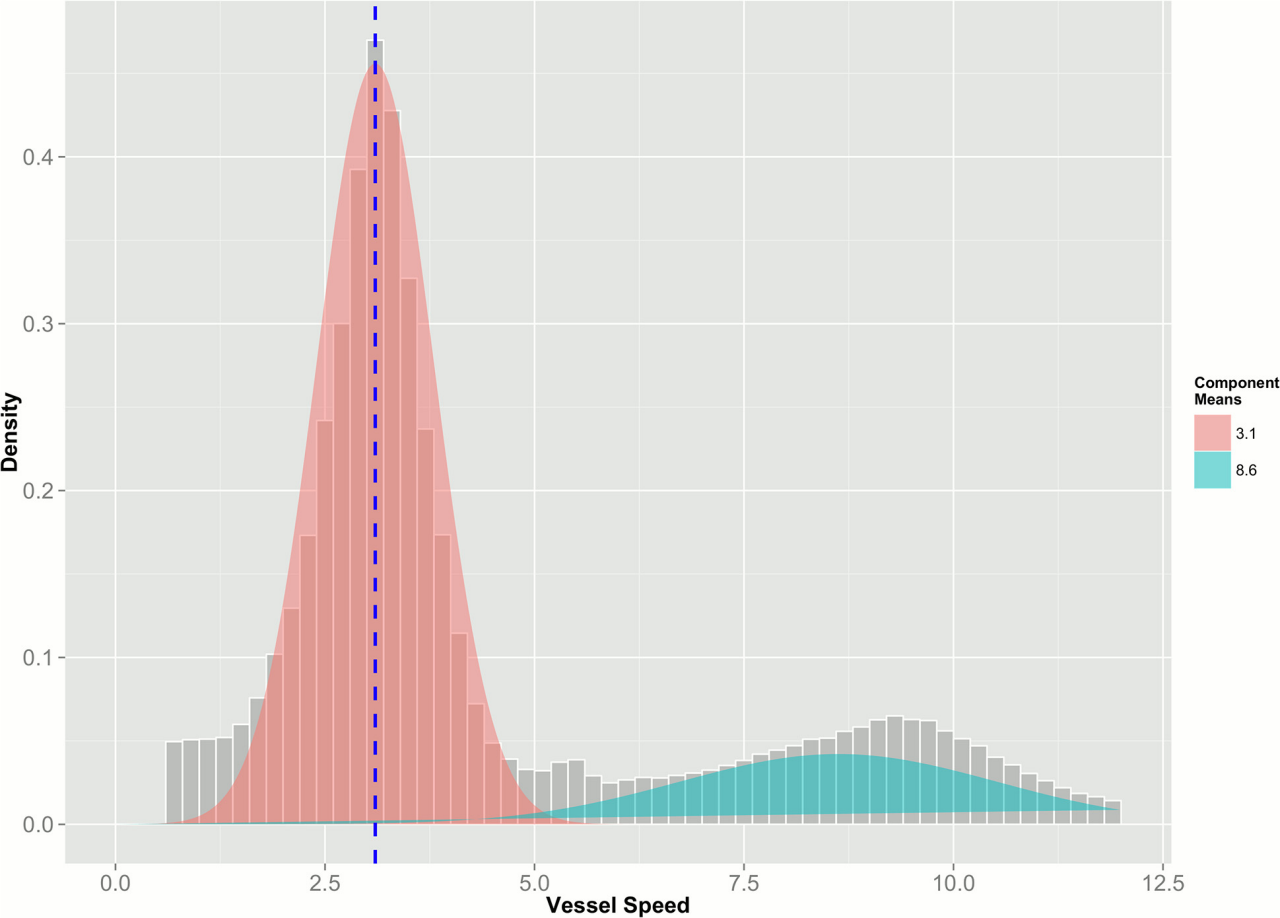
Example of speed profile for one vessel and fitting of a bi-modal distribution through the EM algorithm. The confidence lines at 1.5 standard deviation around the first mode indicate the speed thresholds that were used to classify the AIS messages as fishing. The curve and speed profiles were analysed for each vessel resulting in specific classification threshold on the basis of individual fishing behaviour.

Fig 1 provides a typical example of speed profiles for one vessel to exemplify the methodology. Assuming that the speed profiles are characterized by only two speed modes is plausible especially in light of our data preparation steps: by omitting the messages with a speed of zero, we are filtering out a component in the distribution of speed values. A fourth component related to searching speed was omitted since it was present in very few vessels. Using an Expectation Maximization (EM) algorithm [26, 27] it is possible to provide estimates of the two distributions' parameters and to assign the observations to a particular model component. The EM algorithm has two steps: expectation and maximization. The Expectation step (E-step) aims to estimate the ownership probability, which in other terms is the expected values of the missing data giving the current model estimate. The Maximization (M-step) instead computes the maximum likelihood model parameters given the observed data and the previously calculated expected value of the missing ones. In the case of mixture models the maximization step results in a weighted regression for each model component and mixing components.

The distribution of speed profiles for every vessel was analysed in the statistical software R using the mixtools library and the function NormalmixEMcomp2 specifically designed for two Gaussian mixtures [28].

The final results of the EM algorithm were, for every vessel, a vector containing the parameters of the two Gaussian components: the fishing speed and the steaming speed distributions. For the purpose of the analysis we considered only the fishing speed distribution, i.e. the first mode, and its corresponding parameters.

The speed confidence interval related to fishing activities for a specific vessel was defined at minus/plus 1.5 standard deviations from the first mode.

Despite the speed interval was calculated for every vessel, the value of 1.5 obtained after sensitivity analysis was deemed to be applicable to all the fishing vessels. Accepting a value of 1.5 for the entire fleet could find its justification in the predominance of one type of fishing gear employed – trawl.

### Validation of the fishing activity identification results

The validation of the fishing activity identification method was performed using detailed logbook data of the Swedish fleet in the same period covered by the AIS data. The relevant data from the logbooks included 7,523 fishing trips by a total of 112 vessels (12 vessel of length between 0 and 15 m, 61 between 15 and 24 m, 30 between 24 and 40 and 9 above 40 m).

The use of Swedish logbook data offered the advantage of providing the specific coordinates where the fishing gear is deployed instead of a generic reference to ICES rectangles as in the case of most other EU Member States.

In other studies the validation of fishing identification method for VMS data was performed through on board observers [8]. Albeit being more precise, this approach is resource demanding and can be applied only to a limited number of vessels and trips with no guarantees that the validation can be extended to other categories of vessels and over time.

Logbooks provide a single point measure of the fishing operation corresponding to the coordinates and time when the catch occurred. In order to use logbooks data to validate in binary terms (true positive, false positive, true negative, false negative) a series of AIS messages relating to the fishing operation it would be necessary to define a spatial and/or temporal range. Since the attribution of this range is arbitrary instead of adopting a binary validation scheme, we considered a stochastic validation approach by computing the distance of each AIS message from the fishing grounds of each vessel constructed from the logbooks.

**Fig 2:**
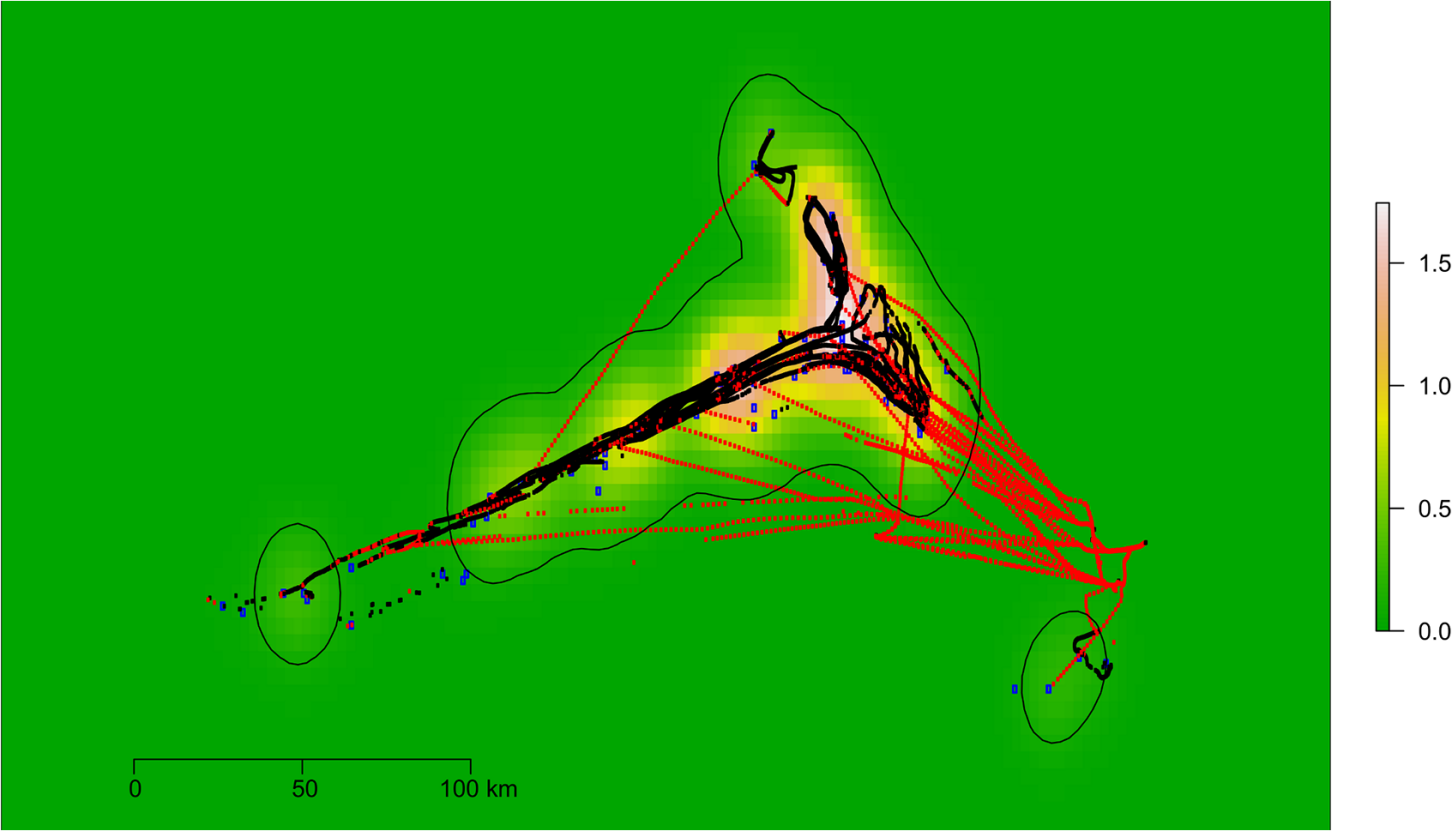
Example of the validation of the method to detect fishing from non-fishing. Blue points correspond to notifications of fishing activity from the logbook data. Points in red represent the position of AIS messages classified as non-fishing, and point in black are messages classified as fishing. The underlying density raster represents a kernel density estimate of the fishing grounds on the basis of the logbook data. Data refers to the fishing activity of a single vessel between January and August 2014.

The coordinates of the fishing operation from the logbooks were used to estimate an utilisation distribution (UD) according to a model in which the use of space can be described by a bivariate probability density function. The UD was estimated with the kernel method developed in [29] and using the R package adehabitarHR [30]. A raster was created from this UD and the values of this raster were associated to each message of the same vessel on the basis of a spatial overlay. Fig 2 gives an example of the validation approach. Each blue point corresponds to a position of a fishing operation from the logbook data. Points in red represent the position of AIS messages classified as non-fishing, and points in black, to messages classified as fishing. The underlying density layer represents the UD estimated from the logbook data. All data refer to the fishing activity of one vessel between January and August 2014.

The values of geographical distance were normalised across vessels and summary statistics were calculated both for the messages classified as fishing and those classified as non-fishing, for the overall fleet, by gear and by single vessel. A paired t test was finally used to see if the validation scores for two groups of fishing and non-fishing were statistically different.

### High resolution fishing effort map

The final step of the analyses was to produce a high resolution fishing effort map for the area of the case study. Since in the data used for the case study AIS messages were regularly spaced at 5 minutes intervals fishing effort was calculated as sum of messages classified as fishing multiplied by 5 minutes and the vessel engine power. The resulting effort values were converted in kW by fishing days and spatially aggregated from the specific coordinates of each AIS message to a grid with cells of 1 by 1 nautical mile.

## Results

**Fig 3:**
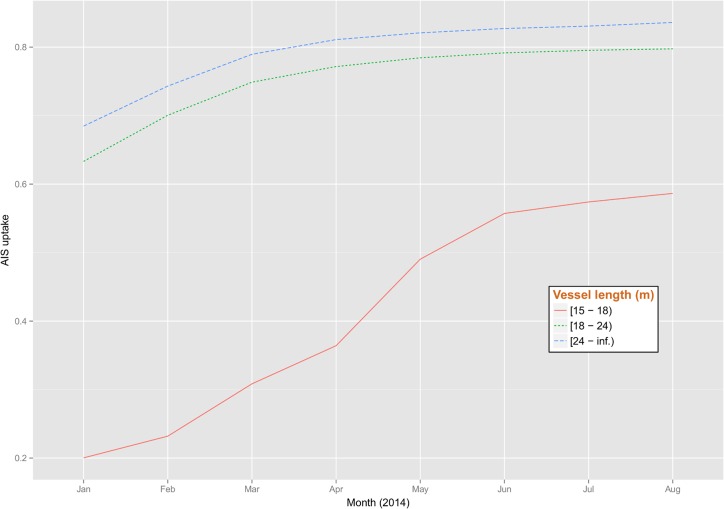
Cumulative level of uptake of the AIS in the EU fleet by three vessel length classes and month. The percentage is calculated as number of vessel which activated the system against the total registered as active in the EU fleet register.

The EU fleet register reported for 2014 a total of 8,130 active vessels above 15 meters in 745 ports. Fig 3 shows the cumulative rate of uptake of the AIS by these vessels by month. The results indicated that overall at least 75% of these vessels above 15 meters adopted the AIS by August 2014. The cumulative rate of adoption shows a steep increase in May 2014 when the obligation to adopt the AIS was extended to vessels above 15 meters of length.

The level of uptake was of 57.4% for vessels between 15 and 18 meters of length (1,195 vessels with AIS out of 2,082 registered vessels), of 79.5% for vessels between 18 and 24 meters of length (2,550 vessels with AIS out of 3,206 registered vessels) and 83.0% for vessels above 24 meters (2,361 vessels with AIS out of 2,842 registered vessels).

**Fig 4:**
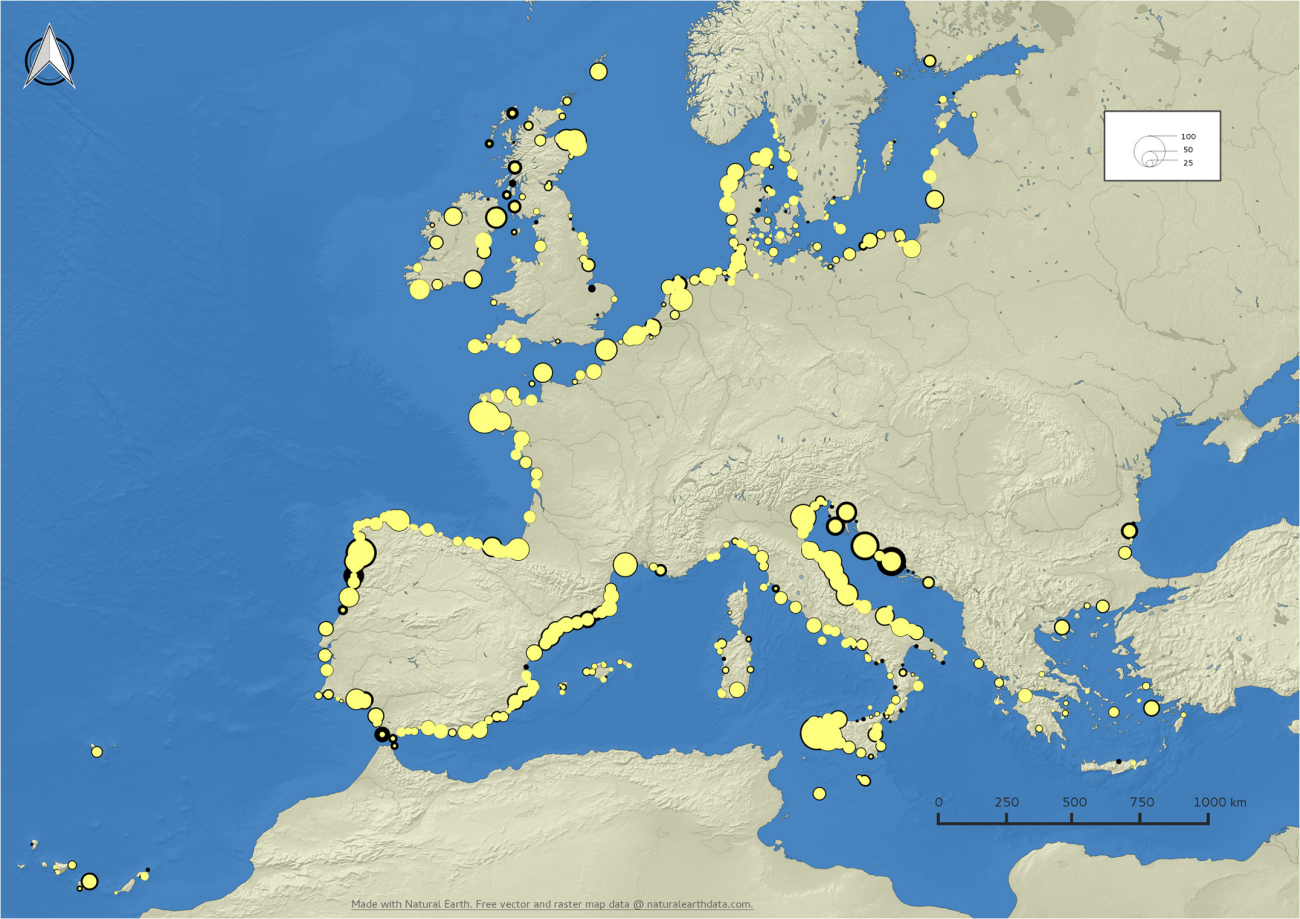
Level of uptake of the AIS in the EU fleet by fishing port. The size of the circles represents the number of vessels above 15 m of length registered in each port and the thickness of their contour line is proportional to the number of vessels for which no AIS message was recorded in the between January and August 2014.

The map in Fig 4 shows the uptake level by fishing port. The size of the symbols is proportional to the number of registered vessels above 15 meters of length while the border is proportional to the numbers of vessels for which it was not possible to retrieve any AIS positioning messages and therefore indicates low rates of uptake.

In 426 EU fishing ports the level uptake of AIS was greater than 75% (4,371 vessels with AIS out of 4,944 registered vessels), in 228 ports it was between 25% and 75% (1,753 vessels with AIS out of 2,818 registered vessels) and in 91 ports it was lower than 25% (30 vessels with AIS out of 368 registered vessels). The level of uptake was lower in some ports along the West Coast of Scotland, in Croatia and in ports along the Atlantic coast of Spain and Portugal.

**Fig 5:**
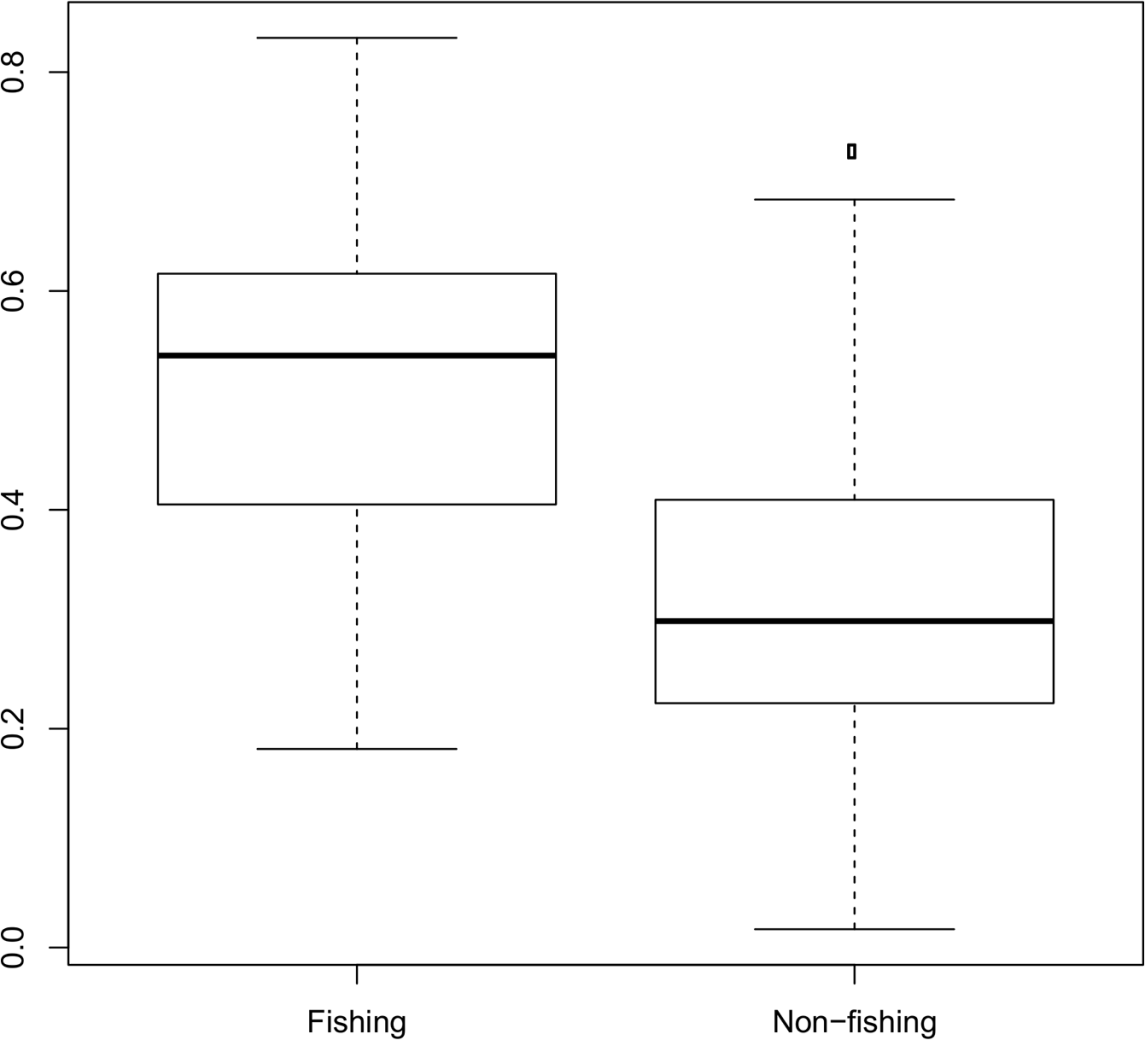
Results of the validation exercise. The two distributions represent the values for messages classified as fishing and those classified as non-fishing in respect of fishing grounds defined for each vessel from the logbook data. Lower values indicate closeness to the historical areas of activity of each vessel.

Fig 5 shows the results of the validation exercise for the entire Swedish fleet. The two distributions are related to the average validation score by vessel for messages classified as fishing and non-fishing. Higher values indicate closeness to the centres of the typical areas of activity. The average validation score by vessel was significantly higher for messages classified as fishing (0.51 ± 0.15) than for messages classified as non-fishing (0.31 ± 0.13) (paired t-test, p-value < 2.2e-16, df = 110).

More detailed statistics by fishing gear and single vessel showed that the performance of the algorithm changed between different gears and individual vessels. In particular the performance resulted higher for otter trawler, bottom and mid-water, pair trawler, purse seiner and Danish seiner and lower in the case of gillnetter, long-liner and fishing pots. At the individual vessel level higher differences in the validation score between fishing and non-fishing and therefore better performance of the classification method were recorded for vessels having more AIS messages available to fit the characteristic bi-modal speed profiles.

**Fig 6:**
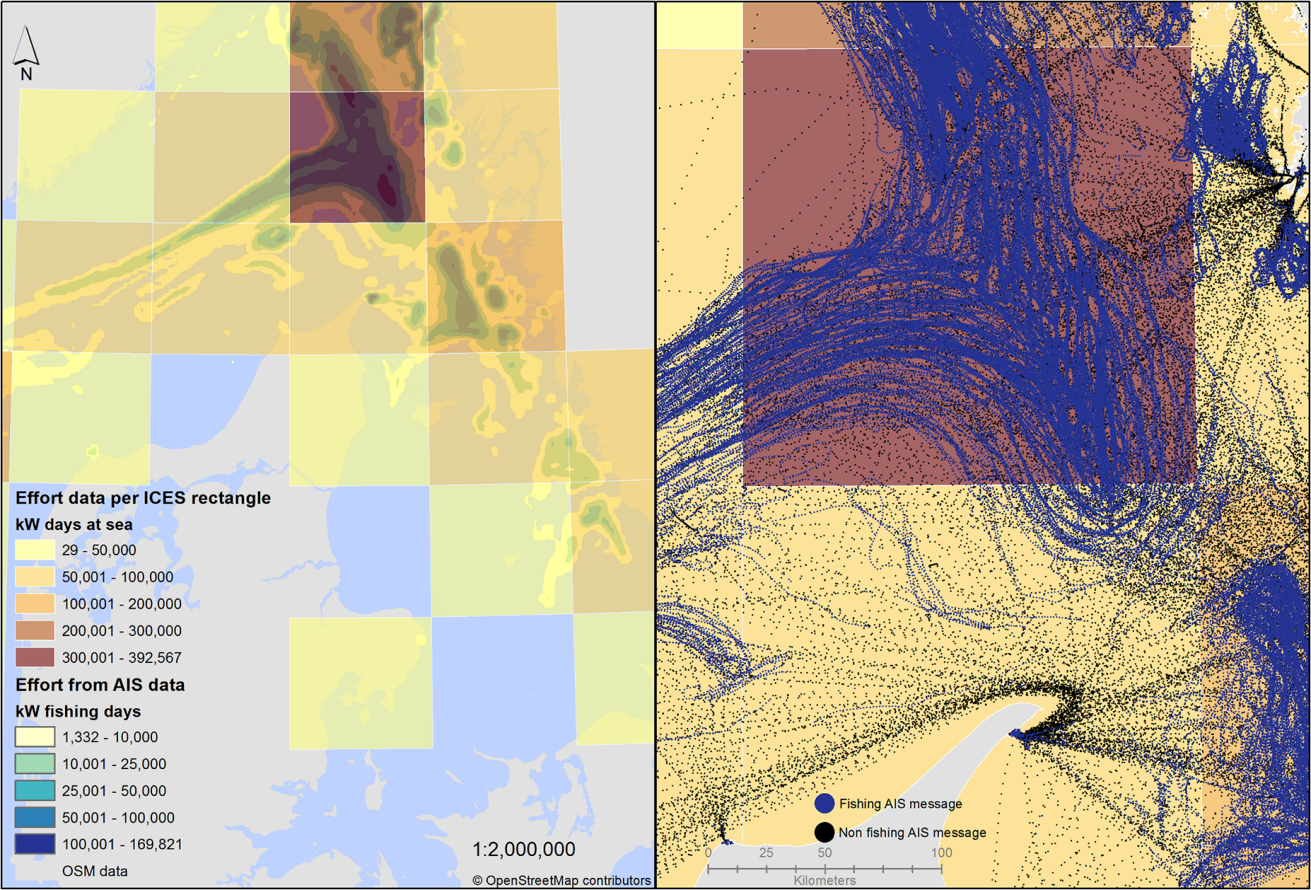
Map of fishing effort calculated from the AIS messages (density layer) and from the logbook data (ICES rectangles).

The map in Fig 6 presents the fishing effort calculated from the AIS vessels of the Swedish fleet in the period January-August 2014. The effort relates to the fishing activity after the exclusion of AIS messages classified as non-fishing.

As a term of comparison the same map shows effort values by ICES rectangles calculated from logbook data for the same group of vessels and same period. The approach adopted for the calculation of effort from the logbooks was to allocate the entire duration of each trip, from departure to arrival in the port, to the ICES rectangle where catches were notified. In case of trips with catches notified in more than one ICES rectangle the duration of the trip was divided in each of them proportionally to the volume of catches. The time was converted into days and multiplied by the engine power to get to the same unit of effort of kW-days used for the AIS data. Since the effort calculated from AIS only refers to fishing activity while the effort from logbooks relates to days at sea it is normal to expect higher absolute values in the case of logbooks. Other differences may derive from misreporting in the logbooks or from poor coverage of the AIS. Despite these differences in magnitude the two approaches produced a very similar spatial distribution.

**Fig 7:**
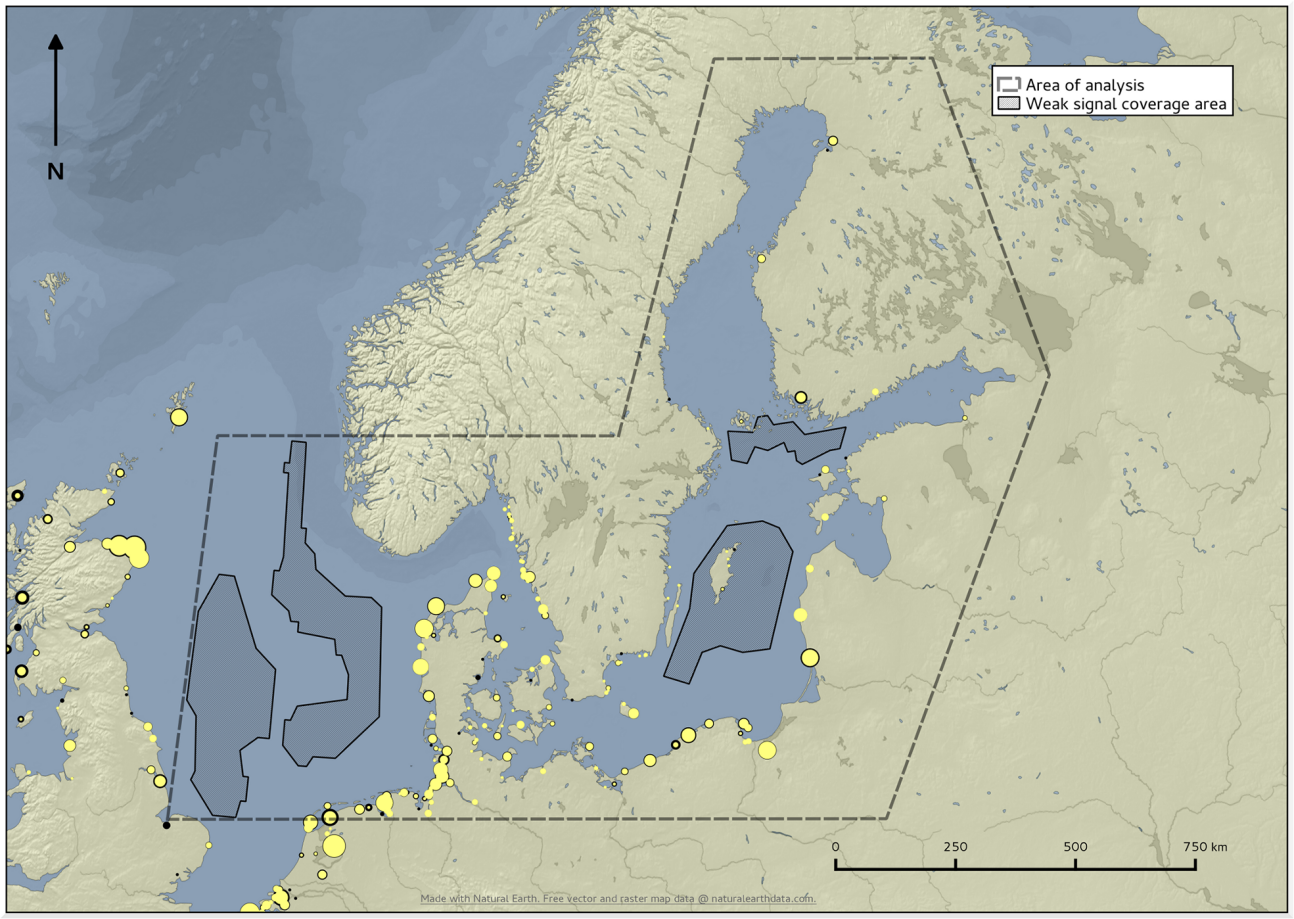
Map of coverage of the AIS signal in the study area.

The final map in Fig 7 shows the geographic extent of the analysis area together with the areas of weak signal coverage. The latter are located in areas distant from the coastline where the VHF propagation can be not optimal. In the west zone we can notice two areas with poor coverage separated by a big area where coverage is good. We can assume that the good coverage area is due to the presence of AIS base stations installed on offshore platforms.

## Discussion

The aim of this study was to assess the level of uptake of the AIS in the EU fishing fleet and to explore the feasibility of producing a European map of fishing effort with high spatial and temporal resolution.

Using the Swedish fleet as case study, a smaller sample of this dataset was used to validate the method to identify fishing activity. From the same dataset a map of fishing effort was produced and accompanied by an assessment of the coverage level of AIS messages.

The level of uptake of AIS in the EU fishing fleet increased considerably from May 2014, when the obligation of adopting the system was extended to vessels of length greater than 15 meters. Although there are some differences between fishing ports, overall the uptake reached in only few months a level of at least 75%. The results of this analysis are subject to AIS data spatial coverage and therefore represent a conservative estimation of uptake and fishing effort figures.

Low levels registered in some ports may be linked to the following factors: problems in finding a correspondence between the fleet register and the AIS, insufficient coverage of the AIS (Croatian ports in the mid Adriatic), the fact that the registered vessel are operating outside EU waters (ports on the Atlantic coast of Spain and Portugal) or the fact that some of the vessels registered in the fleet registered did not have any activity in the reference period. The level of uptake was measured as number of vessels for which at least one message was received. This is indicative that the vessel has adopted the AIS but should not be interpreted as an assessment of the level coverage of activity of the vessels through the AIS.

The literature provides several examples of methods and tools which can be used to identify fishing from non-fishing activity in the analysis of VMS data. In this respect the more frequent refresh rates of the AIS solves the need to estimate intermediate positions between consecutive messages. On the other hand the higher volume of AIS data poses higher computational demands especially considering our long term goal of performing the analysis on an EU scale.

The method we adopted follows the lines indicated by the literature to explore frequency speed profiles of the vessels, with lower speed being associated to towing activities and higher to steaming. The use of EM algorithm to find the parameters of the speed profiles of individual vessels proved to be efficient in computational terms and suitable for the fast processing of large AIS dataset.

Several tests on scalability and computing performance of our methodology were carried out on a bigger AIS dataset using the statistical software R, specifically the mixtools and data.table libraries. The results were comforting: around 2 minutes to estimate fishing and steaming speed for more than 5,000 European fishing vessels.

The method is essentially data driven and using only speed information independently from other contextual information such as the position of the messages in the track or additional variables as bearing and vessel characteristics. The identified thresholds are modelled on each vessel and, in the case there is sufficient data to define characteristic speed profiles, they can be taken as representative of individual fishing behaviour. The results proved to be more reliable in the case of fishing vessels with towed gear and in particular in the case of trawlers. Different approaches would need to be considered in the case of other gears. Vessels with fixed gear for examples, such as pots, have different speed profiles, characterized by the presence of messages with speed of values zero (messages that have been omitted in our model). Further improvements of the model will include zero speed messages that are not located in the port and also considering non Gaussian mixture morel components.

Regardless of the shape of the distributions it is natural to expect that fishing corresponds to the speed with the highest frequency in the speed profiles (first mode) considering that fishermen would tend over the long period to optimise the time spent at sea and the fuel consumption, concentrating great part of their time spent at sea in the fishing activity rather than at steaming. Starting from this consideration a direction which would merit more investigation in future research is to use the analysis of speed profiles as indicator of fishing efficiency. A higher frequency of AIS messages for steaming rather than fishing speed is indicating that a vessel is travelling for longer periods to reach the fishing grounds and has therefore higher cost and fuel consumption for the same amount of effective fishing effort.

The validation step was able to prove that AIS messages classified as fishing are closely related to the typical fishing grounds areas of each vessel. Nonetheless, the validation methodology has an intrinsic limitation since a punctual notification of the fishing operation is used as a reference for the entire extent of the fishing operation. As mentioned in the results this limitation did not allow performing a validation in binary terms. The use of on-board observer would provide more reliable information for the validation step. However the validation with observers is more expensive and, if applied to a limited number of vessels, does not guarantee that the validation results can be extended to other fisheries.

The map in Fig 6 exemplifies the advantages of moving from a relatively low resolution of the ICES rectangles to the more precise assessment of effort through the AIS similarly to what could be achieved with VMS. Several studies indicated that a high aggregation of ICES rectangles is too approximated to assess specific spatial effects of the fishing activity. A precision of 1NM or less is more appropriate in respect of the 30 natutical miles of ICES rectangles for spatial analyses of the environmental impacts of fishing effort [15] [31]. A more accurate calculation of fishing effort is particularly needed when assessing the spatial impact of the fishing activity on the environment as well as the socioeconomic impacts on fisheries from the introduction of marine protected areas.

The comparison between the fishing efforts calculated from the AIS and from logbooks at the aggregated level of ICES rectangles, despite the constant lower values of AIS, showed similar distributions.

The possibility to discriminate fishing from non-fishing has several important implications on how the effort is computed. High precision calculations allow considering and spatially allocating effort as effective fishing time rather than time spent at sea. Currently effort data in the EU is assembled from logbooks using different approaches. The approaches on how time at sea and fishing times are recorded and aggregated can lead to very different estimates of effort across countries and detailed fishing activity data could help addressing these discrepancies.

In addition, more precise effort estimates will allow taking into account the different efficiency levels of the fisheries in fisheries management policies.

While AIS offers a high level of resolution to assess fishing activity in space and time an essential piece of information which is missing is the linkage to the catches and targeted species. For this information the reference to logbooks will still be essential. The use of AIS can compensate for the lack of detailed and reliable logbook information up to a limited extent; given the fact that it is not providing information on catches it will help analysing better the spatial distribution of fishing activity but will not provide a direct link to stocks.

Before moving to a large scale application one aspect which needs further improvements is how to evaluate the coverage of AIS. This is critical in the case of AIS since the technical characteristics of the system and the fact that it was not specifically introduced with fisheries control in mind represent limitations in ensuring a complete and systematic coverage. As shown in Fig 7 the level of coverage may change in different areas and over time. These changes are influenced by the technical characteristics of the AIS signal. The main challenge in assessing coverage is that in areas where vessel estimated trajectories are limited or are absent it cannot be determined whether the absence of vessels trajectories is due to the deficiency of the AIS radio signal strength or to a real absence of traffic routes or activities.

Mapping fishing effort at high spatial-temporal resolution represents a great step forward for spatial analysis of environmental impacts of fishing activity, maritime spatial planning and potentially for fisheries economic studies. Our study showed that AIS is rapidly getting adopted by a large majority of fishing vessel above 15 meters. Intrinsically AIS offers the same data of VMS with an improvement in terms of temporal resolution. The main advantage represented by AIS in respect of VMS lies in the possibility to assemble a large dataset covering all EU waters and performing an assessment of the spatial distribution of fishing effort not only limited at few specific case studies. Issues still to be addressed are related to the evaluation and consideration of the coverage levels and a refinement of methods to identify fishing activity in the case of vessels not using towed gears.
